# Development of Press-Coated, Floating-Pulsatile Drug Delivery of Lisinopril

**DOI:** 10.3797/scipharm.1301-27

**Published:** 2014-04-14

**Authors:** Swati C. Jagdale, Vishnu M. Suryawanshi, Sudhir V. Pandya, Bhanudas S. Kuchekar, Aniruddha R. Chabukswar

**Affiliations:** 1 Department of Pharmaceutics, MAEER’s Maharashtra Institute of Pharmacy, Kothrud, Pune, 411 038, Maharashtra, India.; 2 Quality Assurance, Nulife Pharmaceuticals, Pimpri, 411 018 Pune, Maharashtra, India.; 3 Department of Pharmaceutical Chemistry, MAEER’s Maharashtra Institute of Pharmacy, Kothrud, Pune, 411 038, Maharashtra, India.

**Keywords:** Floating, Press-coated, Carrageenan, Xanthan, Pulsatile, Delivery

## Abstract

Lisinopril is an angiotensin-converting enzyme (ACE) inhibitor, primarily used for the treatment of hypertension, congestive heart failure, and heart attack. It belongs to BCS class III having a half-life of 12 hrs and 25% bioavailability. The purpose of the present work was to develop a press-coated, floating-pulsatile drug delivery system. The core tablet was formulated using the super-disintegrants crosprovidone and croscarmellose sodium. A press-coated tablet (barrier layer) contained the polymer carrageenan, xanthan gum, HPMC K4M, and HPMC K15M. The buoyant layer was optimized with HPMC K100M, sodium bicarbonate, and citric acid. The tablets were evaluated for physical characteristics, floating lag time, swelling index, FTIR, DSC, and *in vitro* and *in vivo* behavior. The 5% superdisintgrant showed good results. The FTIR and DSC study predicted no chemical interactions between the drug and excipients. The formulation containing xanthan gum showed drug retaining abilities, but failed to float. The tablet containing HPMC K15M showed a high swelling index. The lag time for the tablet coated with 200 mg carrageenan was 3±0.1 hrs with 99.99±1.5% drug release; with 140 mg HPMC K4M, the lag time was 3±0.1 hrs with 99.71±1.2% drug release; and with 120 mg HPMC K15M, the lag time was 3±0.2 hrs with 99.98±1.7% drug release. The release mechanism of the tablet followed the Korsmeyer-Peppas equation and a first-order release pattern. Floating and lag time behavior have shown good *in vitro* and *in vivo* correlations.

## Introduction

The pulsatile drug delivery systems are beneficial for drugs having chronopharmacological behavior. These systems are designed according to the circadian rhythm of the body. The principle rationale for the use of pulsatile release is for the drugs where a constant drug release, i.e. a zero-order release, is not desired. The release of the drug as a pulse after a lag time (period of no drug release) has to be designed in such a way that a complete and rapid drug release follows the lag time. The types of pulsatile drug delivery systems are capsular, osmotic, single, and multiple-unit systems based on the use of soluble or erodible polymer coatings, rupturable membranes, pH-induced, and externally regulated systems. Products available as once-daily formulations based on pulsatile release are Pulsincap®, Contin®, and Ciform® [1–6].

The human body has many built-in rhythms known as biological clocks. Broadly, these can be classified as ultradian, circadian, and infradian. Ultradian cycles are shorter than a day, e.g. the time taken for a nerve impulse to be transmitted. Circadian cycles last about 24 hrs, e.g. sleeping and waking patterns. Infradian cycles are longer than a day, e.g. the menstrual cycle [5]. The gastroretentive pulsatile drug delivery system is useful for those drugs which have pH-dependent solubility, poor bioavailability in the gastrointestinal tract (GIT), and a narrow absorption window. These considerations led to the development of oral pulsatile release dosage forms possessing gastric retention capabilities [7–9].

Circadian phase-dependent patterns have been well-documented in conditions such as asthma, arthritis, epilepsy, migraine, allergic rhinitis, cardiovascular disease (myocardial infarction, angina, and stroke), and peptic ulcer disease, with particular times where symptoms are more prominent and exacerbated. Day/night variations in asthmatic dyspnea and variations in the incidence of myocardial infarction occur throughout the early morning hours. Treating these diseases with immediate release dosage forms may be impractical if the symptoms of the disease are pronounced during the night or early morning. Chronotherapeutics refer to a treatment regimen in which *in vivo* drug availability is timed to match the rhythms of disease, in order to optimize therapeutic outcomes and minimize side effects. Pulsatile drug delivery systems can be classified into site-specific systems in which the drug is released after a lag time at the desired site within the intestinal tract (e.g., the colon), or time-controlled devices in which the drug is released after a well-defined time period. Pulsatile systems are therefore known to deliver the drug at the right site of action at the right time and in the right amount, thus providing spatial and temporal delivery and increasing patient compliance [10–13].

Lisinopril is a drug of the angiotensin-converting enzyme (ACE) inhibitor class that is primarily used in the treatment of hypertension, congestive heart failure, and heart attacks. It is also used in preventing the renal and retinal complications of diabetes. The drug has a half-life of 12 hrs. This drug belongs to BCS Class III, having good water solubility. Lisinopril is slowly and incompletely absorbed after oral administration with a bioavailability of 25–30%. The objective of the present study was to improve gastric retention, so consequently, the bioavailability of the drug. The release is expected as a burst, i.e at once after a lag time. The rationale for the development of an appropriate formulation is to provide the drug at the right time, i.e. early morning. The formulation has a rapid release core tablet of lisinopril with superdisintegrants. The core was press-coated by the layer of polymer to impart pulsatile release and finally on the top, a buoyant layer was added for gastric retention. The formulation was evaluated for its properties and correlated with the *in vitro* and *in vivo* buoyancy studies.

## Materials and Methods

### Materials

Lisinopril was a gift sample from Dr. Reddys Lab, Hyderabad. Microcrystalline cellulose (MCC, Avicel PH-102), croscarmellose sodium (Ac-Di-Sol), and **crospovidone (** Polyplasdone XL-10 BASF) were gift samples from Nicolas Piramal Limited, Mumbai. HPMC K4M, HPMC K15M, and xanthan gum were gift samples from Colorcon Asia Pvt Ltd, Mumbai.

### Formulation of Core Tablets by Direct Compression

The inner core tablets were prepared by using the direct compression method. Different preliminary batches of core tablets were taken to fix concentrations of superdisintegrant in the tablets. The concentration of superdisintegrant was varied from 1–5 mg/tablet. As shown in Table 1, the powder mixtures of lisinopril, microcrystalline cellulose, cros-povidone, croscarmellose sodium, and lactose were dry-blended for 20 min followed by the addition of magnesium stearate. The mixtures were then further blended for 10 min. 100 mg of the resultant powder blend was compressed using a rotary tablet machine (Cadmac, Rimek 8 Station Rotary Minipress supplied by Karnavati Pvt. Ltd, Ahmadabad) with a 9-mm punch at a compression pressure of around 90 kg/cm^2^ [14, 15].

### Formulations of the Pulsatile Release Tablet (PRT)

The optimized core tablet was used for the preparation of the pulsatile release tablet. The powder mixture used as an erodible outer shell contained HPMC K4 M, HPMC K15 M, carrageenan, and xanthan gum as shown in tables 2 and 3. Half of the barrier layer material was weighed and transferred into an 11-mm die, then the core tablet was placed at the center, and the remaining half of the barrier layer materiel was added into the die. The tablets were compressed at a pressure of around 300 kg/cm^2^. The same procedure was applied to all of the powders [16].

### Formulations of the Floating-Pulsatile Release Tablet (FPRT)

The floating-pulsatile release tablet was designed to comprise a pulsatile release tablet with a top cover containing a buoyant layer. The buoyant layer included the hydrocolloid gelling agent, HPMC K 100 M, which upon contact with gastric fluid formed a gelatinous mass, sufficient for cohesively binding with a drug release layer. The buoyant layer also included the effervescent components, sodium bicarbonate and citric acid, which were fabricated so that upon arrival in the stomach, carbon dioxide is liberated by the acid present in gastric fluid and entrapped in the gellified hydrocolloid. This produced an upward motion of the dosage form and maintained its buoyancy [17].

### Compositions of the Buoyant Layers

The compositions of the buoyant layers of the FPRT for the floating tablet are shown in Table 4. All powdered excipients were mixed for 5 min with a mortar and pestle to form a homogenous powder mix. Different fillers were used to adjust the tablet weights, and the effects of fillers on floating time were observed. For the preparation of the FPRT tablets, the optimized pulsatile release tablet batches C2, C6, and C9 were used in further studies for compression with the buoyant layers [18].

### Evaluation of the Floating-Pulsatile Release Tablets (FPRT) [19–24]

#### Physical Evaluation Thickness

The thickness of all tablets was measured using a vernier calliper.

#### Hardness Test

The Monsanto hardness tester was used for the determination of the tablets’ hardness. The tablet was placed in contact between the plungers and the handle was pressed. The force of fracture was recorded. The test was carried out as per Indian Pharmacopoeia (IP) 2010 guidelines.

#### Weight Variation

The weight of all the tablets was taken on an electronic balance and the weight variation was calculated. The test was carried out as per Indian Pharmacopoeia (IP) 2010 guidelines.

#### Friability Test

For each formulation, the friability of the tablets was determined using the Roche friabilator. In this test, tablets were subjected to the combined effect of shock abrasion by utilizing a plastic chamber which revolves at a speed of 25 rpm, dropping the tablets to a distance of 6 inches in each revolution. The tablets were then dusted and reweighed. Percent friability (%F) was calculated as follows:

Eq. 1 % F=(loss in weight/initial weight)×100

The test was carried out as per Indian Pharmacopoeia (IP) 2010 guidelines.

### Determination of % Drug Content

The tablets were crushed and powder equivalent to 20 mg of lisinopril was weighed accurately and dissolved in distilled water. The solutions were filtered through a membrane filter (0.45 mm). The drug content was analyzed at 206 nm by a UV spectrophotometer (Varian Cary 100).

### FTIR Study

The floating-pulsatile release tablets (C2+B4, C6+B4, C9+B4) were compressed and powdered. The powder along with potassium bromide was used for the FTIR studies.

### Swelling Index Determination

Tablets were weighed individually (designated as W1) and placed separately in glass beakers containing 200 ml of 0.1 N HCl and incubated at 37°C±1°C. At regular 1-hour time intervals until 10 hrs, the tablets were removed from the beakers, and the excess surface liquid was removed carefully using the paper. The swollen tablets were then reweighed (W2) and the swelling index (SI) was calculated using the following formula:

Eq.2 $$\text{SI}=\frac{\text{W}2-\text{W}1}{\text{W}1}\times 100$$

### In Vitro Buoyancy Determination

The floating behavior of the tablets was determined using the USP dissolution apparatus-II in 900 ml of 0.1 N HCl, which was maintained at 37±0.5°C and rotated at 50 rpm. The floating lag times as well as total floating time were observed.

### Similarity Factor

BIT software was used for the calculation of the similarity factor. The *in vitro* release profile of the marketed Lipril tablets (Lupin) was performed under similar conditions as used for *in vitro* release testing of the test product. The similarity factor between the two formulations (Lipril and formulated formulation) was determined using the data obtained from the drug release studies.

### Differential Scanning Calorimetry (DSC)

Differential scanning calorimetry (DSC) was used to characterize the thermal properties and possibility of any interactions between the drug and excipients. The DSC thermograms were recorded using a differential scanning calorimeter (DSC 823e, Mettler Toledo, Switzerland). Approximately 2–5 mg of each sample were heated in a pierced aluminum pan up to 300°C at a heating rate of 10°C/min under a stream of nitrogen at flow rate of 50 ml/min. Thermal data analyses were carried out.

### Kinetics Modeling of Drug Release

In order to gain insight into the drug release mechanism, the release data were examined for best fitting into zero-order, first-order, and Higuchi’s square root of time mathematical models, the Hixson and Crowell powder dissolution method, and the Korsmeyer and Peppas model. The kinetic modelling was obtained with the help of PCP disso V3 software [25, 26].

### In Vitro Drug Release

The release rate of lisinopril from the floating tablets was determined using the dissolution apparatus II (paddle method). The dissolution test was performed using 900 ml of 0.1 N HCl at 37±0.5°C and 50 rpm. A sample (5 ml) of the solution was withdrawn from the dissolution apparatus hourly for 12 hrs, and the samples were replaced with fresh dissolution medium. The samples were filtered through a 0.45-μm membrane filter and diluted to a suitable concentration with 0.1 N HCl. The absorbance of these solutions was measured at 206 nm using Varian Cary 100 double beam UV spectroscopy.

### In Vivo Studies

The X-ray technique was used to determine the gastric residence time of the tablets. Floating-pulsatile release tablets of the formulations C2+B4, C6+B4, and C9+B4 were selected for *in vivo* gastric residence time studies. The formulations were prepared for *in vivo* studies using barium sulphate as the radiopaque material. For *in vivo* testing, three healthy volunteers were selected. Volunteers were asked to swallow the tablet with sufficient water after a meal under the supervision of a registered doctor. The X-rays of the tablets in the volunteers were recorded at intervals of 1, 2, and 4 hrs [27, 28].

For the *in vivo* tests, the tablets with the following compositions were compressed:

Formulation C2 + Buoyant layer, C6 + Buoyant layer, C9 + Buoyant layer:

For the core tablet: croscarmellose sodium: 5 mg, magnesium stearate: 10 mg, barium sulphate: 20 mg, lactose: 40 mg, microcrystalline cellulose: 25 mg.

Buoyant layer: HPMC K 100M: 130 mg, sodium bicarbonate: 90 mg, citric acid: 70 mg, lactose: 30 mg. Erodible outer shell-carrageenan (100+100) mg, HPMC K4M (70+70) mg, HPMC K15M (60+60) mg.

## Results

### Evaluation of the Core Tablets

The hardness, friability, drug content, and *in vitro* disintegration are shown in Table 5. In all of the formulations, the hardness test indicated good mechanical strength, whereas the friability was less than 1% which indicated that the tablets had good mechanical resistance. The *in vitro* disintegration times for the formulations varied from 14 to 71 seconds. It was observed that when croscarmellose sodium was used as a disintegrant, the tablets were disintegrated within a short time due to the easy and high swelling abilities of croscarmellose sodium as compared to crospovidone. For the development of the pulsatile release study, disintegration time had to be short to obtain the burst effect. The drug content was found to be high (>98.99±0.5) and uniform in all of the tablet formulations. It ranged from 98.99±0.5 to 99.89±0.15 and was uniform in all tablet formulations.

**Fig. 1:**
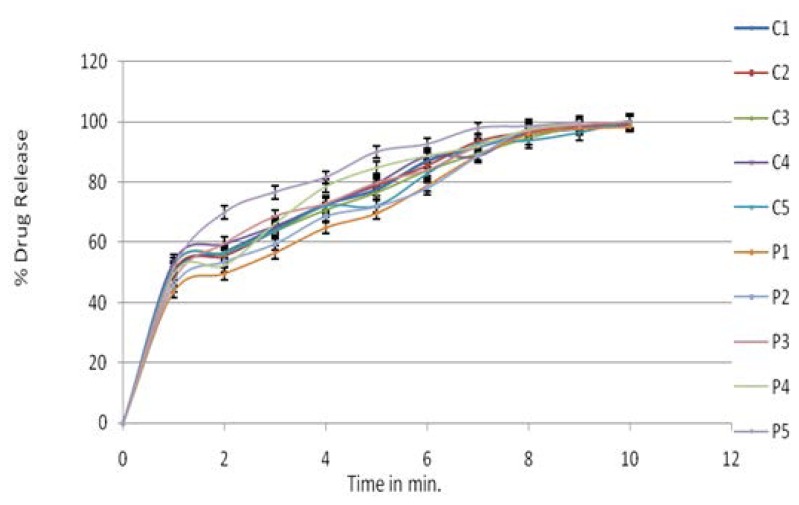
*In vitro* drug release profiles of the core tablet

The rapid increase in the dissolution of lisinopril with an increase in croscarmellose sodium may be attributed to the rapid swelling and disintegration of the tablet. Crospovidone exhibited capillary activity and pronounced hydration with little tendency of gel formation and disintegrated the tablet rapidly, but into larger masses of aggregated particles, later resulting in the slow release of the drug. Formulation S5 showed satisfactory hardness, % drug content, the lowest disintegration time, and high drug release. So S5 was considered as the optimized formulation and it was taken for further studies. The dissolution data for the core tablet are as shown in Fig. 1.

### Evaluation of the Pulsatile Release Tablet (PRT)

**Fig. 2:**
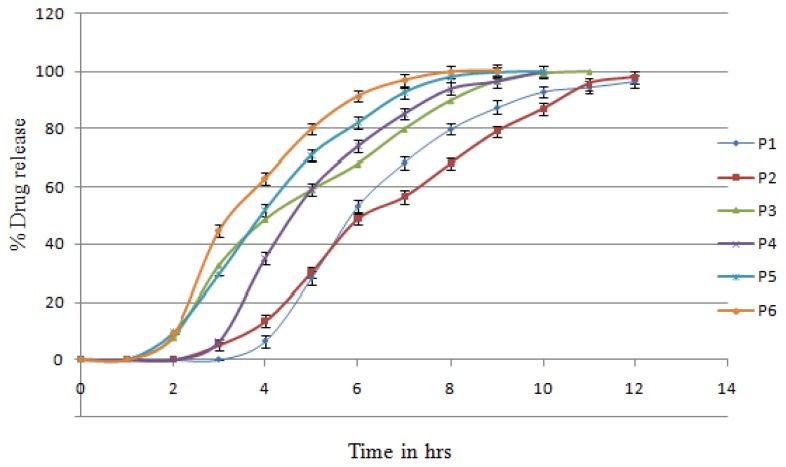
*In vitro* release profiles of batches P1–P6

**Fig. 3:**
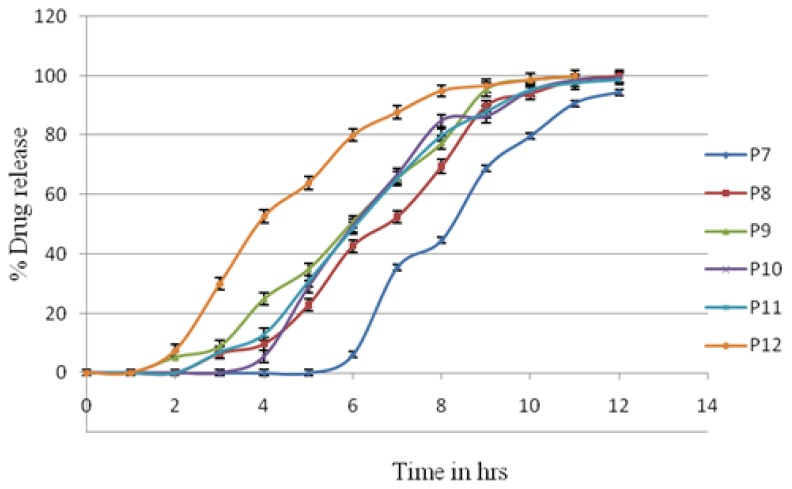
*In vitro* release profiles of batches P7–P12

The core tablet containing crospovidone was compression-coated with carrageenan, HPMC K4, HPMC K15, and xanthan gum. These batches were taken as preliminary batches for the study of individual polymers. The *in vitro* release profiles of lisinopril from the different-coated systems are as shown in figures 2 & 3.

It was observed from figures 2 & 3 that carrageenan showed a lag time of 2 h, which was followed by a sigmoidal release pattern with 100% drug release at the 10^th^ hour. As the concentration of the carrageenan coating increased from P3 to P1, the lag times extended to 3 hrs. This then followed a delayed release profile with 100% drug release at the 11^th^ to 12^th^ hour. Carrageenan, at the highest concentration, also failed to hold the tablet coating (time was max 3 hrs) and have the burst effect. The other possible reason may have been the penetration of media in carrageenan, which led to early bursting of the core tablet in only 3 hrs.

Xanthan gum showed a lag time of 1 hour, resulting in rapid and complete drug release at the 9^th^ hour. The P4 batch contained the highest concentration of gum leading to more lag time than P5 and P6. But this tablet did not maintain its shape throughout dissolution process. The pulsatile release tablet containing xanthan gum was not used for further investigation.

HPMC K4M showed a lag time of 2 hrs and then followed the sigmoidal release pattern with 100% drug release at the 11^th^ hour. As the concentration of the HPMC K4M coating increased, the lag time extended to 5 hrs and then followed the delayed release profile with 100% drug release at the 12^th^ hour.

HPMC K15M showed a lag time of 2.5 hrs and then followed the sigmoidal release pattern with 100% drug release at the 11^th^ hour. As the concentration of the HPMC K15M coating increased, the lag time extended to 3 h and then followed the delayed release profile with 100% drug release at the 12^th^ hour. Both HPMCs clearly indicated the concentration dependence on lag time.

**Fig. 4:**
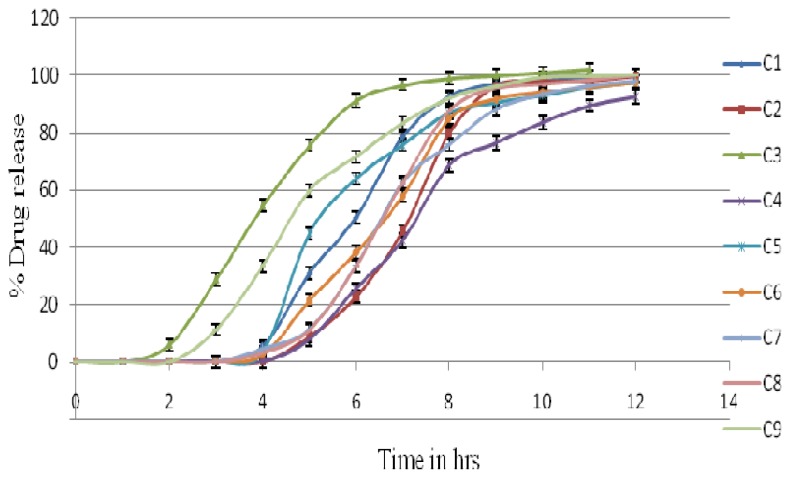
*In vitro* release profiles of batches C1–C9.

Different batches of the pulsatile release tablets of carrageenan, HPMC K4M, and HPMC K15M were prepared using croscarmellose sodium in the core tablet. Fig. 4 shows the drug release pattern of batches C1–C9. From the dissolution profile, it was observed that batch C2 (200 mg), C6 (140 mg), and C9 (120 mg) were optimized batches. C9 was selected as it contains a minimum concentration of polymer HPMC K15 M, i.e 120 mg. To study the effect of the lowest concentration after the development of pulsatile floating delivery, this batch was selected. In the dissolution procedure, the coating layer gradually started to erode up to a limiting thickness. After this stage, a rupture of the shell was observed under the pressure applied by the swelling of the core tablet and lisinopril was released. This pressure was high due to the high swelling property of croscarmellose sodium, which resulted in the burst effect along with complete and rapid drug release. Other batches’ (i.e. C1, C4, C5, C7, and C8) amounts of the coating polymer were too high. In C3, the amount of the coating polymer was too little so that it could not achieve the desired lag time. The drug release clearly depended on the kind and amount of hydrophilic polymer applied on the core [22–24]. The lag time of the tablet coated with 200 mg carrageenan was 3±0.1 hrs with 99.99±1.5% release, for 140 mg HPMC K4M, the lag time was 3±0.1 hrs with 99.71±1.2% release, and with 120 mg HPMC K15M, the lag time was 3±0.2 hrs with 99.98±1.7% release. After this, the % drug release remained constant due to the non-maintenance of the sinking condition. So formulations C2, C6, and C9 were considered as optimized batches and were taken for further studies. [27]

### Evaluation of Floating-Pulsatile Release Tablets

Only the floating-pulsatile release tablets of the optimized batches (C3+B4, C6+B4, and C9+B4) were evaluated for the friability test, hardness test, and drug content (Table 6).

#### Thickness

The thickness of all the formulations was observed within the range of 4.3±0.3 mm.

#### Swelling Index Determination

**Fig. 5:**
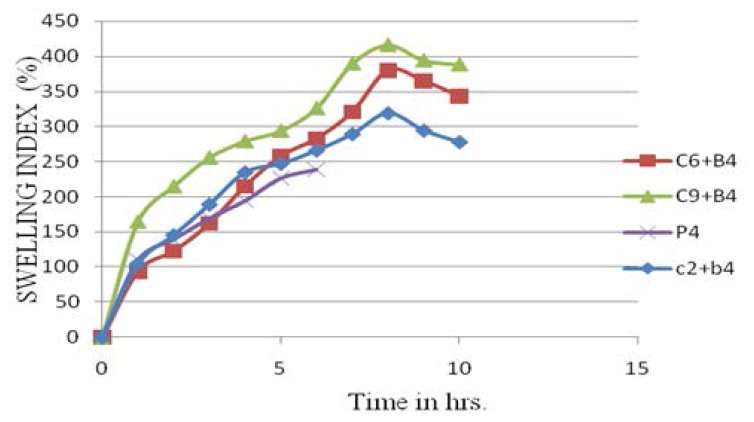
Swelling Index of Lisinopril

Tablets containing HPMC K15M showed the highest swelling index as compared to carrageenan and HPMC K4M. The tablet containing xanthan gum showed swelling, but after some time the tablet could not maintain its shape and integrity. Carrageenan, HPMC K4M, and HPMC K15M showed a constant increase in the swelling index up to 7 hrs, after this there was a decrease due to the start of tablet erosion (Fig. 5).

### In Vitro Drug Release of the Floating-Pulsatile Release Tablet

The FPRT formulation consisted of the buoyant layer B4 (Table 4) combined with a PRT-containing core tablet and coated with 200 mg of carrageenan (formulation C2), 140 mg of HPMC K 4M (formulation C6), and 120 mg of HPMC K15M (formulation C9).

**Fig. 6:**
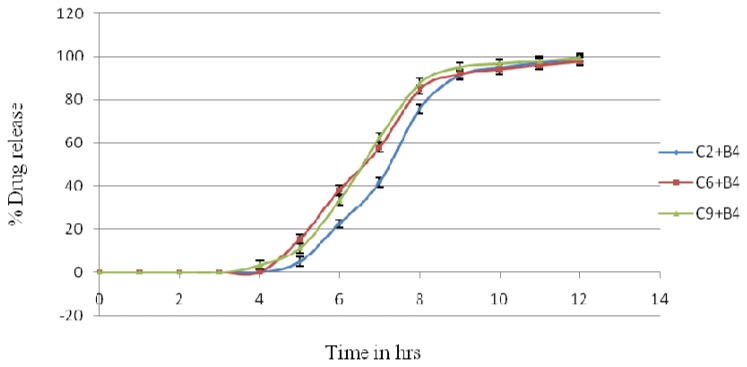
*In vitro* release profiles of batches C2+B4, C6+B4, and C9+B4

The lag time of FPRT coated with 200 mg carrageenan was 4±0.1 hrs, for 140 mg HPMC K4M, it was 4±0.1 hrs, and for 120 mg HPMC K15M, it was 3±0.1 hrs. These were considered suitable lag times for lisinopril in preventing the time-related occurrence of ischemia, because taking the tablet at night will release the drug in the early morning, and based on the half-life of the drug it will remain in the plasma when needed (Fig. 6).

### FTIR Study

The IR spectrum of lisinopril is characterized by the absorption of the C=O group at 1652 cm^−1^. In the spectra of formulations C2+B4, C6+B4, and C9+B4, the band had the same absorption pattern as that of the pure drug, which can be predicted that there may not be any chemical interaction of the drug and excipients [26].

### Differential Scanning Calorimetry

**Fig. 7:**
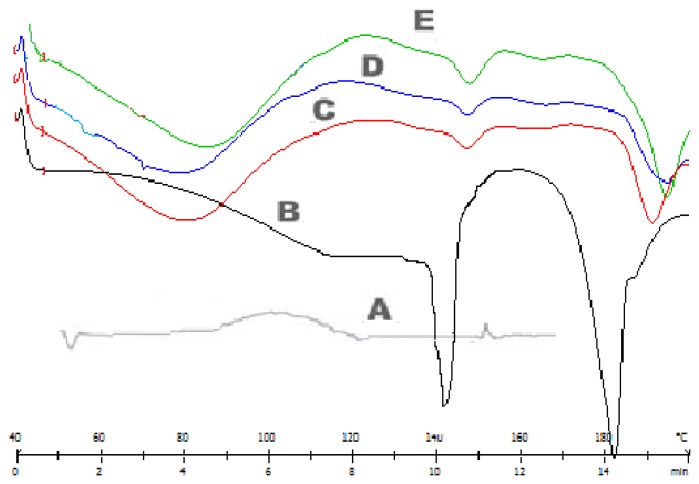
DSC curves of lisinopril and formulations (C2+B4, C6+B4, C9+B4). A: HPMC, B: lisinopril, C: batches C2+B4, D: batches C9+B4, E: batches C6+B4

Figure 7 shows the DSC thermographs of pure lisinopril and formulations C2, C6, and C9. The thermographs obtained by the DSC studies revealed that the melting point of the pure drug is 146–148°C and that of the drug in the formulations is close to 146–148°C. It was concluded that the drug is in the same pure state even in the formulation without interacting with the polymers [26].

### In Vivo Studies

**Fig. 8:**
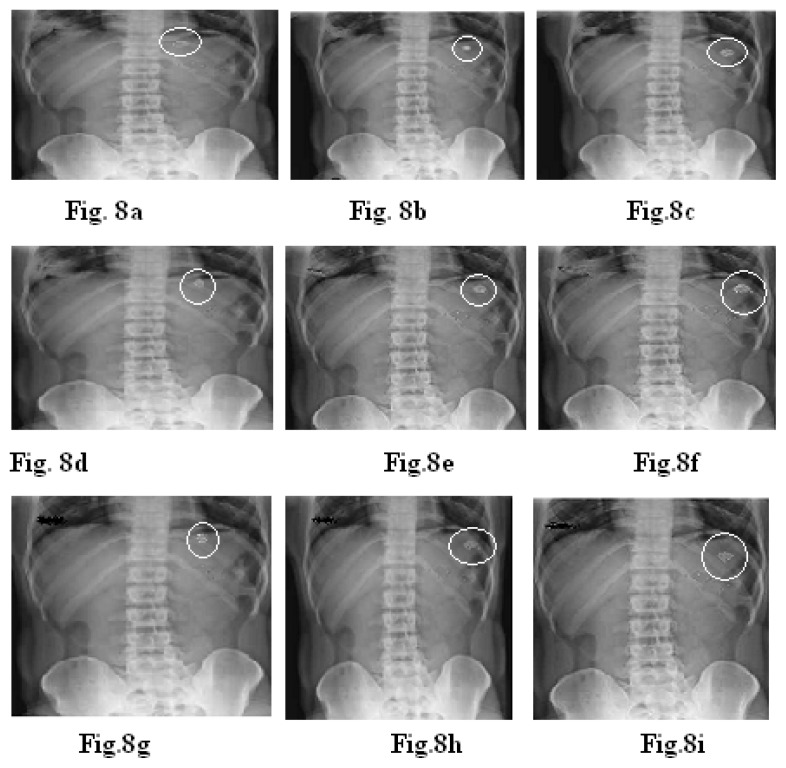
X-rays indicating the position of the floating tablet in the gastrointestinal tract of volunteers for the formulations C2+B4, C6+B4, and C9+B4. X-ray taken at 15 min (a,d,g), at 2 hrs (b,e,h), and at 4 hrs (c,f,i).

Figure 8a shows an X-ray taken at 15 min after administration of the tablet (C2+B4). The tablet can be seen in the stomach. Fig. 8b taken at 2 hrs shows a change in the position of the tablet; this shows that the tablet did not adhere to gastric mucous. Fig. 8c taken at 4 hr shows that the lag time will be completed soon and within a short time interval, the burst effect will be obtained [22, 23].

Figure 8d shows an X-ray taken at 15 min after administration of the tablet (C6+B4). The tablet can be seen in the stomach. Fig. 8e taken at 2 hrs shows a change in the position of the tablet; this shows that the tablet did not adhere to gastric mucous. Fig. 8f taken at 4 hrs shows that the lag time will be completed soon and within a short time interval, the burst effect will be obtained.

Figure 8g shows an X-ray taken at 15 min after administration of the tablet (C9+B4). The tablet can be seen in the stomach. Fig. 9h taken at 2 hrs shows a change in the position of the tablet; this shows that the tablet did not adhere to gastric mucous. Fig. 8i taken at 3 hrs shows that the lag time will be completed soon and within a short time interval, the burst effect will be obtained.

This indicated that the floating ability and bursting effect in the case of the i*n vivo* studies were similar to the *in vitro* studies.

## Discussion

Disintegration time of the core tablet was decreased with increased concentration of croscarmellose sodium and crospovidone. As the concentration of the polymer coating increased, lag time increased. Xanthan gum failed in the development of a successful pulsatile drug delivery system as it could not give a sufficient lag time and was unable to maintain its shape. The other three polymers (carrageenan, HPMC K4M, and HPMC K15M) were used to further develop effective pulsatile drug delivery systems.

### In Vitro Buoyancy Determination

The floating lag times as well as total floating time were observed. The floating behavior of the tablets depended on added fillers in the buoyant layer. The buoyancy lag time was less than 1 min only for formulations containing 130 mg HPMC K100M, 90 mg sodium bicarbonate, 70 mg citric acid, and 30 mg lactose. The floating time was more than 12 hrs.

### Kinetics Modeling of Drug Release

Different results that were obtained and the model which best fits the drug release from the different formulations are shown in Table 7. The Korsmeyer-Peppas model indicated that the release mechanism was not well-known or that more than one type of release phenomena was involved as Fickian diffusion (Higuchi Matrix), anomalous transport, and zero-order release, whereas first-order release indicated the dependence on the concentration remaining in the dosage form. This model is applicable to study the release profiles of the pharmaceutical dosage forms such as those containing water-soluble drugs in porous matrices.

### Similarity Factor

Similarity factor analysis between the formulations C2+B4, C6+B4, C9+B4, and Lipril (Lupin) showed an *f*_2_ less than 50, which confirmed the no-similarity in the drug release of both the formulation and marketed, because the marketed preparation was immediate release and the formulations C2+B4, C6+B4, C9+B4 were pulsatile release, which was expected to give a burst release after lag time.

## Conclusion

The lag time of drug release from the FPRT formulation can be readily modulated by varying the concentration of carrageenan, HPMC K4M, and HPMC K15M in the outer barrier layer. Good correlation was observed between the *in vitro* and *in vivo* performance of the floating-pulsatile release tablet, suggesting that the robust and reliable ability to produce a lag time before drug release may make this formulation useful as a chronopharmaceutical drug delivery system. It can be considered as one of the promising formulation techniques for preparing floating-pulsatile drug release systems of lisinopril.

## Figures and Tables

**Tab. 1:** Formulations of core tablets

Ingredients	S1	S2	S3	S4	S5	S6	S7	S8	S9	S10
Lisinopril	20	20	20	20	20	20	20	20	20	20
Croscarmellose sodium	1	2	3	4	5	–	–	–	–	–
Crosprovidone	–	–	–	–	–	1	2	3	4	5
Mg-stearate	10	10	10	10	10	10	10	10	10	10
Microcrystalline cellulose	25	25	25	25	25	25	25	25	25	25
Lactose	44	43	42	41	40	44	43	42	41	40
TOTAL (mg)	100	100	100	100	100	100	100	100	100	100

**Tab. 2:** Formulations for the pulsatile release tablet containing crospovidone

Sr. No.	Ingredients	Formulation Codes											

		P1	P2	P3	P4	P5	P6	P7	P8	P9	P10	P11	P12
1	Carrageenan	220	200	180	–	–	–	–	–	–	–	–	–
2	Xanthan gum	–	–	–	200	180	160	–	–	–	–	–	–
3	HPMC K4 M	–	–	–	–	–	–	180	160	140	–	–	–
4	HPMC K15 M	–	–	–	–	–	–	–	–	–	160	140	120

**Tab. 3:** Formulations for the pulsatile release tablet containing croscarmellose sodium

Sr. No.	Ingredients	Formulation Codes								

		C1	C2	C3	C4	C5	C6	C7	C8	C9
1	Carrageenan	220	200	180	–	–	–	–	–	–
2	HPMC K4 M	–	–	–	180	160	140	–	–	–
3	HPMC K15 M	–	–	–	–	–	–	160	140	120

**Tab. 4:** Compositions of the buoyant layers

No.	Ingredients	Formulation codes				

		B1	B2	B3	B4	B5
1	HPMC K 100M	140	150	160	130	160
2	Sodium bicarbonate	50	60	70	90	90
3	Citric acid	50	50	60	70	80
4	Dicalcium phosphate	–	20	–	–	–
5	Microcrystalline cellulose	20	–	–	–	–
6	Lactose	–	–	30	30	30

**Tab. 5:** Evaluation of core tablets

Formulation	Hardness (kg/cm^2^)	Friability (%)	%Drug content	Disintegration time (sec.)
S1	3.6±0.17	0.61±0.10	98.99±0.3	67.4±1.2
S2	3.7±0.11	0.67±0.11	99.29±0.7	38.5±1.8
S3	3.3±0.15	0.65±0.11	99.45±0.11	42.1±1.3
S4	3.8±0.11	0.63±0.15	99.75±0.15	30.2±1.5
S5	3.5±0.12	0.62±0.09	99.89±0.12	14.4±1
S6	3.2±0.10	0.60±0.16	99.25±0.13	71.2±1.5
S7	3.9±0.08	0.62±0.13	99.76±0.11	69.3±1.7
S8	3.5±0.16	0.62±0.16	99.69±0.10	52.8±1.2
S9	3.6±0.15	0.63±0.11	99.88±0.09	48.6±1.4
S10	3.8±0.14	0.62±0.12	99.92±0.11	38.7±1.3

**Tab. 6:** Evaluation of floating and pulsatile release tablets

Sr. No.	Formulations	Hardness (kg/cm^2^)	Friability (%)	% Drug content
1	C2+B4	3.9±0.16	0.45±0.13	99.24±1.1
2	C6+B4	4.3±0.18	0.48±0.11	99.48±2.2
3	C9+4	4.0±0.14	0.56±0.48	99.56±3.1

**Tab. 7:** Kinetic modeling of floating–pulsatile release tablets

Formulation	Zero-order plot		First-order plot		Korsmeyer–Peppas plot		Matrix (Higuchi) plot		Hixson–Crowell plot	

	*R*^2^	*k*	*R*^2^	*k*	*R*^2^	*k*	*R*^2^	*k*	*R*^2^	*k*

C2+B4	0.917	7.697	0.987	−0.153	0.996*	18.046	0.788	22.626	0.877	−0.063
C6+B4	0.936	8.021	0.991	−0.161	0.991*	17.231	0.826	23.901	0.903	−0.061
C9+B4	0.922	8.103	0.986*	−0.187	0.923	23.156	0.807	24.045	0.904	−0.069

* …best fitting model.

## References

[1] Sharma GS, Srikanth MV, Uhumwangho MU, Phani Kumar KS, Ramana Murthy KV (2010). Recent trends in pulsatile drug delivery systems - A review. Int J Drug Del.

[2] Reddy RJ, Veera M, Jyothsna T, Mohamed SS, Madhu S, Chetty C (2009). Review On: Pulsatile Drug Delivery Systems. J Pharm Sci Res.

[3] Lalwani A, Santani DD (2007). Pulsatile drug delivery systems - A review. Ind J Pharm Sci.

[4] Belgamwar VS, Gaikwad MV, Patil GB, Surana S (2008). Pulsatile drug delivery systems – review. Asian J Pharm.

[5] Hrushesky WJM (1994). Timing is everything. The Sciences.

[6] Salunkhe AK, Dias RJ, Mali KK, Mahajan NS, Ghorpade VS (2011). Formulation and evaluation of floating pulsatile drug delivery system of metoprolol tartrate. Der Pharmacia Letter.

[7] Shidhye SS, Lotlikar VM, Ghule AM, Phutare PK, Kadam VJ (2010). Pulsatile delivery systems: A Approach for chronotherapeutic Diseases. Syst Rev Pharm.

[8] Arora S, Ali J, Ahuja A, Baboota S, Qurashi J (2010). Pulsatile drug delivery systems A approach for controlled drug delivery. Ind J Pharm Sci.

[9] Savani HD, Turakhiya J, Patel J, Goyani M, Akbari B (2013). Floating pulsatile drug delivery system: a review. Universal J Pharm.

[10] Lin S, Kawashima Y (2012). Current status and approaches to developing press coated chronodelivery drug systems. J Control Rel.

[11] Jain D, Raturi R, Jain V (2011). Recent technologies in pulsatile drug delivery systems review. Biomatte.

[12] Jagdale SC, Sali MS, Barhate AL, Kuchekar BS, Chabukswar AR (2013). Formulation, development, and evaluation of floating pulsatile drug delivery system of atenolol. PDA J Pharm Sci Tech.

[13] Maroni A, Zema L, Dorly M, Loreti G (2010). Oral pulsatile delivery Rationale and chronopharmaceutical formulations. Int J Pharm.

[14] Janugade BU, Patil SS, Patil SV, Lade PD (2009). Formulation and evaluation of press-coated montelukast sodium tablets for pulsatile drug delivery system. Int J Chem Tech Res.

[15] Ghimire M, McInnes FJ, Watson DG, Mullen AB, Stevens HNE (2007). In-vitro/*in-vivo* correlation of pulsatile drug release from press-coated tablet formulations: A pharmacoscintigraphic study in the beagle dog. Eur J Pharm Biopharm.

[16] Fukui E, Miyamura N, Yoneyama T, Kobayashi M (2001). Drug release from and mechanical properties of press-coated tablets with hydroxyl propyl methylcellulose acetate succinate and plasticizers in the outer shell. Int J Pharm.

[17] Efentakis M, Koligliati S, Vlachou M (2006). Design and evaluation of a dry coated drug delivery system with an impermeable cup, swellable top layer and pulsatile release. Int J Pharm.

[18] Krogel I, Bodmeier R (1999). Floating or pulsatile drug delivery systems based on coated effervescent cores. Int J Pharm.

[19] Badve SS, Sher P, Korde A, Pawar AP (2007). Development of hollow/porous calcium pectinate beads for floating-pulsatile drug delivery. Eur J Pharm Biopharm.

[20] Waterman KC, Fergione MB (2003). Press-coating of immediate release powders onto coated controlled release tablets with adhesives. J Control Rel.

[21] Moon A, Kondawar M, Shah R (2011). Formulation and evaluation of press -coated indomethacin tablets for pulsatile drug delivery system. J Pharm Res.

[22] Chaturvedi K, Umadevi S, Vaghani S (2010). Floating Matrix Dosage Form for Propranolol Hydrochloride Based on Gas Formation Technique: Development and *In-vitro*. Evaluation Sci Pharm.

[23] Jivani RR, Patel CN, Jivani NP (2009). Design and Development of a Self Correcting Monolithic Gastroretentive Tablet of Baclofen. Sci Pharm.

[24] (2010). Government of India, Ministry of health and welfare.

[25] Singh B, Ahuja N, Jain NK (2001). Progress in controlled and Novel Drug Delivery Systems.

[26] Jagdale SC, Ghorpade SA, Kuchekar BS, Chabukswar AR (2011). Novel approach for optimisation of gastroretentive formulation. Drug Deliv Lett.

[27] Jagdale SC, Agavekar AJ, Pandya SV, Kuchekar BS, Chabukswar AR (2009). Formulation and evaluation of gastroretentive drug delivery system of propranolol hydrochloride. AAPS Pharma Sci Tech.

[28] Baumgartner S, Kristl J, Vrecer F, Vodopivec P, Zorko B (2000). Optimisation of floating matrix tablets and evaluation of their gastric residence time. Int J Pharm.

[29] Satwara RS, Patel PK (2012). Formulation and optimization of chronomodulated press-coated tablet of carvedilol by Box–Behnken statistical design. ChronoPhysiol Ther.

[30] Zilpe CR, Dhumale AJ, Gudalwar BR, kawtikwar PS, Sakarkar DM (2012). Development and evaluation of pulsatile press coated tablet to control early morning BP surge. Int J Pharma World Res.

[31] Sibel BP, Suba BR, Vural I, Unlu N, Capan Y (2011). Evaluation of drug-excipient interaction in the formulation of celecoxib tablets. Acta Polon Pharm.

[32] Moon A, Kondawar M, Shah R (2011). Formulation and evaluation of press-coated indomethacin tablets for pulsatile drug delivery system. J Pharm Res.

